# One Step Assembly of Thin Films of Carbon Nanotubes on Screen Printed Interface for Electrochemical Aptasensing of Breast Cancer Biomarker

**DOI:** 10.3390/s16101651

**Published:** 2016-10-06

**Authors:** Muhammad Azhar Hayat Nawaz, Sajid Rauf, Gaelle Catanante, Mian Hasnain Nawaz, Gilvanda Nunes, Jean Louis Marty, Akhtar Hayat

**Affiliations:** 1 Interdisciplinary Research Centre in Biomedical Materials (IRCBM), COMSATS Institute of Information Technology, Lahore 54000, Pakistan; azharhayat@ciitlahore.edu.pk (M.A.H.N.); sajidrauf@ciitlahore.edu.pk (S.R.); mhnawaz@ciitlahore.edu.pk (M.H.N.); 2 BAE: Biocapteurs-Analyses-Environnement, Universite de Perpignan Via Domitia, 52 Avenue Paul Alduy, Perpignan Cedex 66860, France; gaelle.catanante@univ-perp.fr; 3 Technological Chemistry Department, Federal University of Maranhão, CCET/UFMA, Av. Portugueses, Cidade Universitária do Canga, São Luis 65080-040, MA, Brazil; gilvanda.nunes@hotmail.com

**Keywords:** thin films, carbon nanotubes, high density, electrochemical aptasensor, cancer diagnosis

## Abstract

Thin films of organic moiety functionalized carbon nanotubes (CNTs) from a very well-dispersed aqueous solution were designed on a screen printed transducer surface through a single step directed assembly methodology. Very high density of CNTs was obtained on the screen printed electrode surface, with the formation of a thin and uniform layer on transducer substrate. Functionalized CNTs were characterized by X-ray diffraction spectroscopy (XRD), Fourier transform infrared spectroscopy (FTIR), thermogravimetric analysis (TGA) and Brunauer–Emmett–Teller (BET) surface area analyzer methodologies, while CNT coated screen printed transducer platform was analyzed by scanning electron microscopy (SEM), atomic force microscopy (AFM), cyclic voltammetry (CV) and electrochemical impedance spectroscopy (EIS). The proposed methodology makes use of a minimum amount of CNTs and toxic solvents, and is successfully demonstrated to form thin films over macroscopic areas of screen printed carbon transducer surface. The CNT coated screen printed transducer surface was integrated in the fabrication of electrochemical aptasensors for breast cancer biomarker analysis. This CNT coated platform can be applied to immobilize enzymes, antibodies and DNA in the construction of biosensor for a broad spectrum of applications.

## 1. Introduction

Carbon nanomaterials have become the subject of intense research in the last few decades due to their unique structural and physical properties. They are being aggressively exploited to meet many of the current and future needs in the fields of energy, computing, security, life sciences and healthcare. In fact, more and more new carbon materials continue to be discovered and are artificially prepared [1,2]. Among them, carbon nanotubes (CNTs) [3] is an interesting group of carbon materials grabbing attention nowadays due to their unique optical, electronic, and mechanical properties, along with chemical integrity [4]. However, a big challenge for the scientists working in the carbon nanomaterials field is the mass production of structurally homogeneous and pure samples with limited control on solid supports for their integration into different devices. In this work, we present a method for the fabrication of high density thin films of CNTs for selective coating of solid substrates without any prior modification of surface. As a proof of concept, the direct assembly of CNTs was used to fabricate thin films over macroscopic areas of screen printed electrodes and subsequently employed in the construction of electrochemical aptasensor for breast biomarker analysis.

High sensitive and real-time monitoring of single molecule processes based on biomolecular recognition in biological samples is an area of great interest in the field of biomedical science [5,6,7]. Recent (bio) sensing research has witnessed a large number of CNT based transducer methodologies for analysis of DNA, viruses, antigens, disease markers, and whole cells. One of the major issues with CNTs for promising applications is the uniform coating of activated CNTs on the transducer surface because CNTs tend to aggregate into bundles through strong attractive interactions. Pristine CNTs have highly hydrophobic surfaces and are insoluble in almost all solvents, which greatly obstruct their capacity of forming uniform and stable films [8,9,10]. Many chemical strategies, either covalent modification or those based on surfactants, have been reported to functionalize CNTs for propose of long-term solubility and ability to anchor surface selective thin films on a wide range of substrate materials [3,11,12,13]. However, these methods for CNT thin film fabrication may undergo one or several of the following issues: (1) thin film fabrication structure cannot be controlled and results in the disorder of multilayers that are poorly conducting, posing significant barriers to electron transfer in redox reactions, and limits their integration in electrochemical biosensors [14]; (2) requirement of substrate surface modification prior to CNT thin layer assembly with an increase in the number of fabrication steps leading to irreproducible results; (3) difficulty in obtaining surface selectivity to form complex shapes, and getting density of thin films that is too low by using a large amount of CNT/solvent are serious factors that restrict their use in practical applications [15]. CNTs functionalized with organic moieties in a covalent fashion can be optioned for selective assembly of CNTs from solutions onto solid substrates such as transducer platforms in sensing applications. Such a phenomenon has been used in the past for selective coating of HfO_2_ over SiO_2_ surfaces [15].

In this work, we present a thin film assembly method of covalently functionalized CNTs on screen printed carbon electrodes in the construction of an electrochemical aptasensor. CNTs were functionalized by employing diazonium salt chemistry, which resulted in CNTs bearing benzoic acid as an organic moiety, and, subsequently, films were fabricated on carbon electrodes using well-dispersed functionalized CNT aqueous solution. This assembly method offers various potential advantages in the construction of biosensors such as no need of any prior chemical modification of carbon electrode surface, use of water as solvent, one step simple fabrication directly from solution using a very small amount of CNTs and reuse of solution used for thin film assembly. Fabrication of a highly selective monolayer with high density of CNTs in small features with complex shapes on screen printed transducers was expected to provide an ideal platform for on-surface chemistry, and thus has been demonstrated in this work. These modified screen printed carbon electrodes were further exploited to develop a very sensitive electrochemical DNA aptamer-based biosensor to detect mucin (MUC1), a prevalent gene associated with breast cancer [16]. Breast cancer is the most common malignancy among women and the leading cause of cancer mortality worldwide [17]. Early diagnosis and timely therapy could be the most effective ways to improve the survival rate at present. There are also other serum markers that are linked to breast cancer and may be used in clinical practice, including BRCA1, BRCA2, and CA 27.29 carcinoembryonic antigen (CEA), polypeptide antigen (TPA), cytokeratin 19 fragment (CIFRA-21-1), tissue polypeptide specific antigen (TPS), human epidermal growth factor receptor 2 (s-HER2), platelet-derived growth factor (PDGF), vascular endothelial growth factor (VEGF) and osteopontin (OPN) [18,19]. In recent years, a new class of cancer biomarkers has been identified as miRNAs, which is successfully exploited to screen cancer at early stages [20,21]. miRNAs exhibit abnormal levels of expressions during breast cancer [22]. In literature, a number of aptasensors have been reported which have been used for detection of breast cancer biomarkers. Recently, highly sensitive label free electrochemical detection of VEGF has been reported by Shamsipur et al. using an anti-VEGF165 aptamer immobilized on a composite glassy carbon electrode [23]. Some other research groups have also devised electrochemical biosensors for VEGF detection [24,25,26]. A direct aptamer based detection of osteopontin in serum samples has been reported by Cao et al. [27]. Different strategies also have been developed for electrochemical detection of a PDGF biomarker that can be applied to monitor breast cancer tumor progression [28,29,30,31,32,33,34].

MUC1 is used clinically most often to monitor patient cancer therapy at al1 (I–IV) stages [35]. In normal conditions, the MUC1 gene encodes transmembrane mucin proteins, but in breast carcinomas, the MUC1 protein is not restrained to transmembrane, but rather upregulated over the cell surface with more exposed peptide epitopes due to shortened O-glycans [36,37,38,39]. As a result, the level of MUC1 increases in blood circulation, making serum assays potentially useful in tumor detection [40,41]. A biosensor based detection and assessment of concentration of the MUC1 is a direct measure of disease severity [38,42]. Although specific application of the designed surface is demonstrated in the construction of a electrochemical aptasensor, this methodology can be very easily extended to design other types of bioreceptor surfaces such as those employing enzymes, antibodies, or cells as recognition elements.

## 2. Experimental Details

### 2.1. Materials

Sodium phosphate dibasic Na_2_HPO_4_, potassium phosphate monobasic KH_2_PO_4_, magnesium chloride (MgCl_2_), potassium chloride (KCl), sulfuric acid (H_2_SO_4_, 98%), acetone (99%), sodium chloride (NaCl), sodium nitrite (NaNO_2_), potassium ferrocyanide (K_4_[Fe(CN)_6_]), potassium ferricyanide (K_3_[Fe(CN)_6_]), 4-aminobenzoic acid, sodium nitrate, fetal bovine serum, human serum and bovine serum albumin were purchased from Sigma (Taufkirchen, Germany). Lysozyme was from Carbosynth (Berkshire, UK). N-(3-dimethylaminopropyle)-N-ethyle-carbodiimide hydrochloride (EDC) was obtained from Alfa Aesar (Heysham, UK), while cancer antigen mucine MUC1 (25 kU) was purchased from Leebio (Maryland Heights, MO, USA). Multi walled carbon nanotubes (MWCNs) were received from Aldrich. NH_2_ modified aptamer was synthesized and provided by Microsynth, Balgach, Switzerland.

The sequence of the aptamer was as follows:

(5′ GCA GTT GAT CCT TTG GAT ACC CTG G3′)-NH_2._

MUC1 and its aptamer solutions were prepared in binding buffer (BB pH 7.4) containing 1 mM MgCl_2_, 140 mM NaCl, 2.7 mM KCl, 0.1 mM Na2HPO4 and 1.8 mM, KH_2_PO_4_. All solutions were prepared in deionized water from ELGA PURELAB^®^ Ultra water deionizer (High Wycombe, UK).

### 2.2. Apparatus

Fourier transform Infrared (FTIR) spectra of multi-walled carbon nanotubes (MWCNTs) and functionalized MWCNTs were recorded using a Thermo Fisher Scientific Nicolet 6700 spectrometer (Waltham, MA, USA), Brunauer–Emmett–Teller (BET) surface area was analyzed by using Micromeritics Tristar II surface and porosity analyzer (GA, USA), Thermogravimetric analysis (TGA) was performed in a TA Instruments SDT Q 600 (New Castle, DE, USA) from 50 to 800 °C under nitrogen atmosphere (heating rate: 10 °C/min), X-ray diffraction patterns were recorded using a PANalytical Xpert Powder Diffractometer (Almelo, The Netherlands). Scanning electron microscope (SEM) studies were performed at VEGA 3 TESCAN variable pressure mode (LMU) version (Brno, Czech Republic). Images were taken in different magnification ranges at an accelerated voltage of 20 kV, atomic force microscopy was performed by a Park xe-7 atomic force microscope (Suwon, Korea) and electrochemical measurements were performed on a Gamry Reference 3000 potentiostat/galvanostat (Warminster, PA, USA).

### 2.3. Functionalization of Carbon Nanotubes (CNTs)

To perform the functionalization process, 3 mg of MWCNTs were sonicated with a probe sonicator in 10 mL orthodichlorobenzene for 15 min. Afterwards, 4-aminobenzoic acid (45 mg) dispersed in 5 mL acetonitrile was added to the CNT dispersion and the mixture was sparged with nitrogen for 15 min. Sodium nitrite (76.5 mg) was added to the dispersion mixture and the mixture was heated to 60 °C overnight while connected to a vacuum line. CNTs obtained were purified by reprecipitation from an excess of acetone followed by centrifugation at 3.5 K for 30 min. This process was repeated twice and the resulting CNT residue was dried and resonicated in water with a probe sonicator to obtain an aqueous dispersion of functionalized CNTs. These functionalized CNTs remained well-dispersed for period of months.

### 2.4. Thin Film Assembly of CNTs on SPCE

Prior to thin film assembly, screen printed carbon electrodes SPCE were subjected to electrochemical pretreatment by applying several potential cycles between 1 and −1.5 V/pseudo Ag reference electrode with 100 mVs^−1^ scan rate in mixture of 0.5 M H_2_SO_4_ solution until the cyclic voltammetry CV characteristic for a clean SPCE surface was obtained. After electrochemical cleaning, SPCE were exposed to aqueous solution of functionalized CNTs for 1 h, after which the SPCE were removed from solution and rinsed thoroughly with copious amounts of water to remove non-specifically bound CNTs. The SPCE were then carefully dried at room temperature and were used directly to perform the experiments or stored at room temperature for an extended period of time.

### 2.5. Immobilization of Aptamer on CNTs Modified SPCE

The terminal benzoic acid groups on SPCE surface were activated by immersing the SPCE into a solution of 100 mM N-(3-dimethylaminopropyl)-N'-ethylcarbodiimide hydrochloride (EDC) in PBS (pH 7.4) for 60 min. After washing the electrode surface with water, 50 μL of MUC1 aptamer solution at an optimized concentration of 2 μM were incubated onto the activated SPCE surface for 45 min. After incubation, the electrode was rinsed with distilled water to remove unbound aptamer, and, subsequently, the modified SPCE was incubated with 100 μL of 1% Bovine serum albumin (BSA) solution for 60 min to deactivate the remaining terminal groups and block unreacted sites. The MUC1 aptamer modified SPCEs can be used directly as aptasensor, or stored dry at 4 °C for several days without any decrease in the sensitivity.

### 2.6. Electrochemical Impedimetric Measurement

Electrochemical measurements were performed on Gamry Reference 3000 potentiostat/galvanostat. SPCEs were fabricated using a DEK 248 screen-printing system. The SPCE consists of a conventional three electrode configuration with graphite as working (4 mm diameter disk) and counter (16 mm × 1.5 mm curved line) electrodes, and Ag/AgCl (16 mm × 1.5 mm straight line) as pseudo reference electrode. Impedance experiments were carried out at an applied potential of 0.1 V (vs. Ag/AgCl reference electrode) obtained from the redox potential of [Fe(CN)6]^4−/3−^ with a frequency range of 100 kHz–0.2 Hz, an AC amplitude of 10 mV and a sampling rate of 10 points. The Electrochemical Impedance Spectroscopy (EIS) spectra were plotted in the form of complex plane diagrams (Nyquist plots, −Z_im_ vs. Z_re_) and fitted to a theoretical curve corresponding to the equivalent circuit with frequency response analyzer software (FRA) software (Switzerland). For reproducibility of results from different electrodes, ∆ ratio was calculated for each electrode using the following equations [43]:

∆_ratio_ = ∆_s_/∆_p_, ∆_s_ = R_et (Apt-mucine)_,

∆_p_ = R_et (aptamer)_,

where R_et (aptamer)_ was the electron transfer resistance value obtained after aptamer immobilization on the electrode, and R_et (Apt-mucine)_ was the electron transfer resistance value observed after incubation with target analyte mucine.

### 2.7. Analyte Detection, Interference Study and Real Sample Analysis

For MUC1 detection, 50 μL of varying concentrations of MUC1 solutions were incubated on to MUC1 aptamer modified SPCE for 45 min. Interference studies were performed using BSA, lysozyme and fetal bovine serum as possible interfering analytes. Electrochemical experiments were performed in the same way as described for MUC1 detection. The practical applicability of the electrochemical aptasensor was demonstrated by analyzing the analyte in human serum samples.

## 3. Results and Discussion

**Figure 1:**
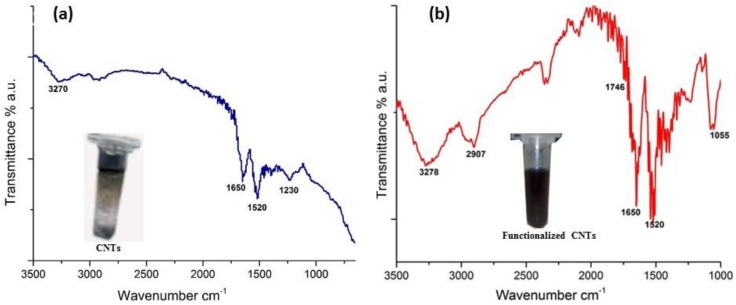
Fourier transform infrared (FTIR) spectra of pure multi-walled carbon nanotubes (MWCNTs) (**a**) and functionalized MWCNTs (**b**), insets shows the dispersion of both in water.

The surface characteristics of the CNTs were determined by N2 adsorption–desorption at 77 K. The BET surface area of MWCNT was found to be 180.1175 m^2^/g, with pore volume of 0.494737 cm^3^/g and pore size of 109.8700 Å. FTIR was used to ascertain the groups attached onto the functionalized CNTs. FTIR spectra of the pure MWCNTs, and functionalized MWCNTs, were measured and shown in Figure 1. Figure 1a shows the FTIR spectra of pure MWCNTs. Here, the peak that appeared at 1520 cm^−1^ is assigned to the characteristics backbone C=C skeletal stretching of CNTs, while the absorption bands at 1650 cm^−1^ and 1230 cm^−1^ are associated with C–C vibrations and C–O stretching vibration, corresponding to the internal defects of carbon nanotubes. The absorption band at 3270 cm^−1^ is more likely from the O–H vibration, associated with amorphous carbon [44].

Figure 1b shows the FTIR spectra of functionalized MWCNTs. The bands at 3278 cm^−1^ and 1746 cm^−1^ can be attributed to O–H/–OH bonds and C=O stretching bonds, respectively. An absorption peak at 1055 cm^−1^ is due to C–O stretching. Hence, it is conjectured that after functionalization of MWCNTs, hydrophilic carboxyl (–COOH) groups are attached onto the MWCNTs. An absorption band at 2907 cm^−1^ corresponds to C–H stretching, indicating an increase in the number of sp^3^ carbon bonds. These bonds are assumed to originate from the breakage of C=C bonds during functionalization [45]. The remaining peaks are the same as described for pure MWCNTs. The insets of Figure 1a,b provide a dispersion comparison of the pure and functionalized MWCNTs. As can be seen from the inset of Figure 1b, the functionalization process resulted in well-dispersed MWCNTs while pure CNTs tend to aggregate in the aqueous medium (Figure 1b)

TGA was used to investigate the presence of functional moieties on the surface of CNTs. From Figure S1, the TGA curve for functionalized CNTs (curve b) indicated an obvious weight loss with a sudden dip when the temperature was increased from 300 °C to 400 °C, which can be attributed to the decomposition of C–O [46]. Figure S2 shows the X-ray diffraction patterns of pure MWCNTs (a) and functionalized MWCNTs (b). MWCNTs exhibited diffraction peaks (2θ) at 25.98° and 43.3°, which can be assigned to the hexagonal graphite structure (0 0 2) and (0 0 1), respectively. The 2θ peaks corresponding to (0 0 2) reflection planes are known as interlayered spacing between adjacent graphite layers, respectively. The (0 0 2) reflection peaks were observed at the same 2θ values in both pure and functionalized MWCNTs diffractions. It can be concluded from the XRD patterns that functionalized MWCNTs retained the same cylinder wall structure and interplanar spacing after the functionalization process. Thus, the structure of MWCNTs is protected after the functionalization processes was confirmed from XRD analysis.

**Figure 2:**
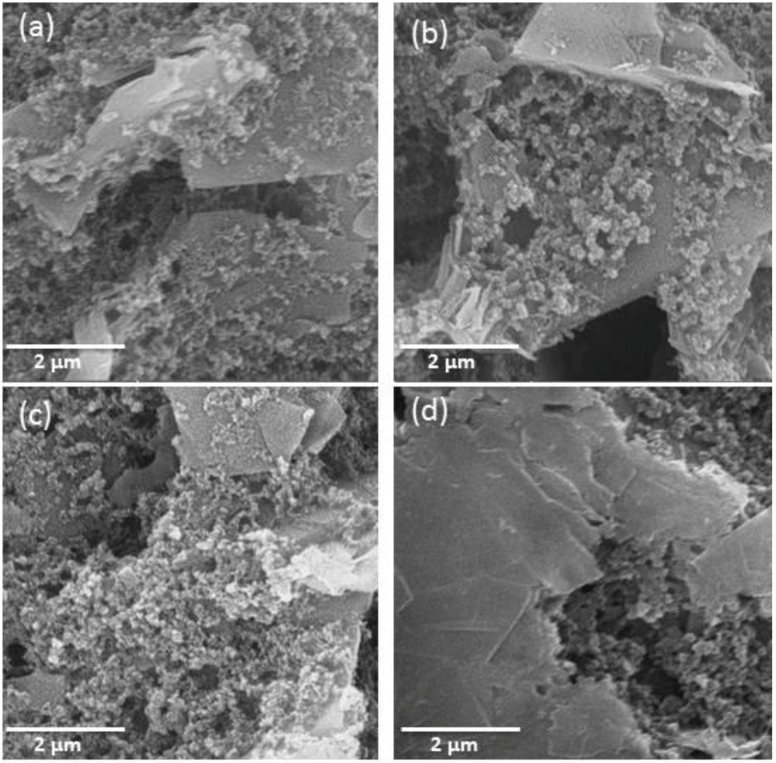
Scanning electron microscope (SEM) images of bare-screen printed carbon electrode (SPCE) (**a**) MWNT-SPCE (**b**), aptamer immobilized MWCNT-SPCE (**c**) mucine–aptamer MWCNT-SPCE; and (**d**) Scale bar: 2.0 μm (**a**–**d**).

The SEM images of the screen printed carbon electrodes at different preparation stages are displayed in Figure 2. It can be seen from the figure that the surface of MWCNT functionalized SPCE has different morphology as compared to the bare electrode with spherical grains of MWCNTs distributed among the carbon particles in the form of threads. These thread like structures showed good density with some porosity and making electrode surfaces more uniform as compared to bare SPCE. The transducer surface was covered with cloudy clusters following incubation with aptamer and mucine.

**Figure 3:**
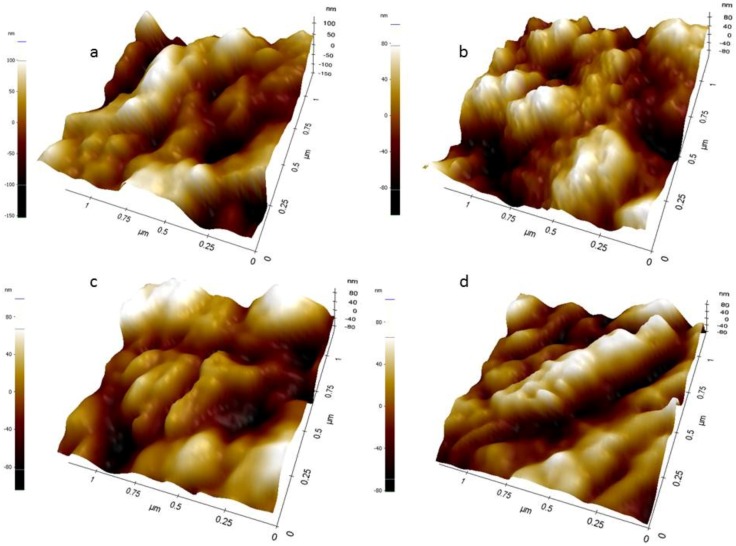
Tapping mode atomic force microscopy (AFM) images of bare-SPCE (**a**); MWNT-SPCE (**b**); aptamer immobilized MWCNT-SPCE (**c**); and mucine–aptamer MWCNT-SPCE (**d**); scale bar: 1.0 μm (**a**–**d**).

Figure 3a–d shows the atomic force microscopy (AFM) topographic surface analysis for bare-SPCE (a) MWCNTs-SPCE (b), aptamer immobilized MWCNT-SPCE (C) and mucine–aptamer MWCNT-SPCE (d). Phase differences between the samples can be explained from surface roughness, and texture for each fabrication step. It can be seen in Figure 3b that MWCNT modified SPE has regular surface patterns (forest) due to the MWCNT thin layer making the surface smoother as compared to the bare electrode Figure 3a. Additionally, calculated values of surface roughness for bare SPE (0.0512 sq. μm) and MWCNT modified SPE (0.0424 sq. μm) demonstrates the difference between the two fabrication steps. The attachment of biomolecules resulted in a change in the forest like morphology of MWCNTs to “rolling hill” like morphology.

### 3.1. Electrochemical Characterization of the Aptasensor

**Figure 4:**
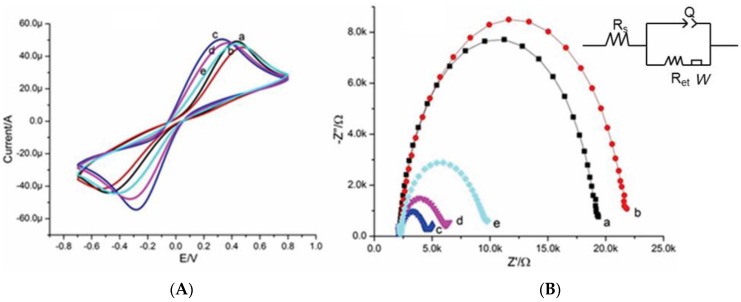
(**A**) cyclic voltammograms of 2.0 mM [Fe(CN)6]4-/3-probe at scan rate of 100 mV/s and (**B**) Nyquist plots for (a) bare SPCE, (b) SPCE/MWCNT modified electrode, (c) SPCE/MWCNT/N-(3-dimethylaminopropyle)-N-ethyle-carbodiimide (EDC) activated modified electrode, (d) SPCE/MWCNT/EDC activated/Apt modified electrode, and (e) SPCE/MWCNT/EDC activated/Apt/Mucine modified electrode.

The electrochemical characterization of the aptasensor surface was evaluated at each fabrication step using CV and EIS in the presence of [Fe(CN)_6_]^4−/3−^ as a redox probe. The presence of redox probe facilitates the electron transfer process, providing high current response from electrochemically inert solution. A low concentration of redox probe [Fe(CN)_6_]^4−/3−^ (2 mM) prepared in binding buffer was used in all electrochemical measurements to avoid its toxic effects towards biomolecules [47]. Peak-to-peak separation and change-in-peak current in voltammograms for modified electrodes are responses conveying information about electron transfer rate constant and resistance to electron transfer. Figure 4a shows the CV for bare SPCE electrode (a); functionalized MWCNTs modified electrode (b); functionalized MWCNTs/EDC activated modified electrode (c); functionalized MWCNTs/Mucine-aptamer modified electrode (d); and functionalized MWCNT/Mucine-aptamer/Mucine modified electrode (e). The bare SPCE electrode showed a characteristic quasi-reversible redox peak having 0.9 V peak-to-peak separation with anodic and cathodic current peak ratio of approximately one. After formation of the MWCNT thin layer, the electron transfer resistance (R_et_) between the redox probe and electrode surface was increased. This increase in R_et_ was attributed to a physical barrier offered by MWCNTs and the presence of negatively charged terminal carboxylic groups COO^−^, resulting in an increased peak-to-peak distance. When negatively charged terminal groups were activated using EDC, R_et_ between the redox probe and electrode surface was decreased to a large extent along with a decrease in the peak-to-peak separation due to electrostatic attraction between a positively/neutrally charged ester intermediate and [Fe(CN)_6_]^4−/3−^ probe. When the mucine–aptamer was immobilized onto the electrode surface, a negatively charged interface was developed due to the phosphate backbone of the aptamer that repels a negatively charged redox probe, resulting in an increased R_et_ value. Binding of mucine onto the immobilized aptamer was characterized with an increase in R_et_.

EIS is also a very effective electrochemical characterization technique for characterizing surface modifications. The MWCNT thin layer formation on SPE and immobilization of biomolecules were conformed through EIS measurements. The Nyquist plot with a semicircle portion at higher frequencies corresponds to the electron transfer resistance and a straight line represents the diffusion resistance. Impedance spectra (Nyquist plots) for each surface modification step are shown in Figure 4b. The interface is modeled using the Randles equivalent circuit with ohmic electrolyte resistance (Rs), the electron-transfer resistance (R_et_), the Warburg impedance element (Zw) resulting from the diffusion of ions from the bulk of the electrolyte to the interface, and the constant phase element (Figure 4b inset) (Q) [48]. The R_et_ depends on the insulating feature at the electrode/electrolyte interface and represents facial properties of the surface. R_et_ is the most directive and delicate parameter to evaluate interfacial properties [49,50]. Therefore, R_et_ was selected among all other circuit elements to get signals for changes on the electrode interface at each fabrication step for the proposed aptasensor. It can be seen that the R_et_ value of the bare SPCE (7.5 kΏ) (curve a) remarkably increased when the electrode was modified with MWCNT thin film (8.3 kΏ) (curve b), which can be attributed to negatively charged terminal carboxylic groups COO^−^ of thin film hampering electron transfer kinetics between the redox probe and the electrode due to electrostatic repulsion of [Fe(CN)6]^4−/3−^ and COO^−^ [51] The terminal carboxylic groups were activated by EDC, resulting in a very large decrease in R_et_ (1 kΏ) (curve c) due to formation of positively/neutrally charged ester intermediate, which promoted the electron transfer from [Fe(CN)6]^4−/3−^ to the electrode surface [52,53]. Afterward, when mucine specific aptamer was covalently anchored on the MWCNT modified electrode, the Ret increased with an increase in the semicircular diameter (1.5 kΏ) (curve d) because of a negative charge from the phosphate backbone of the aptamer. When the aptasensor was used to detect the mucine, R_et_ significantly increases (3 kΏ) (curve e) due to an additional negative charge by the ionization of the carboxylic moieties in the mucine molecule at neutral pH. The prominent changes in the Ret value upon incubation of the target analyte on the aptamer modified transducer surface enable the fabrication of the electrochemical impedimetric aptasensor. The fitting values of the equivalent circuit parameters for various steps of the aptasensor fabrication and the complex formation between aptamer and target analyte were shown in Table S1.

**Figure 5:**
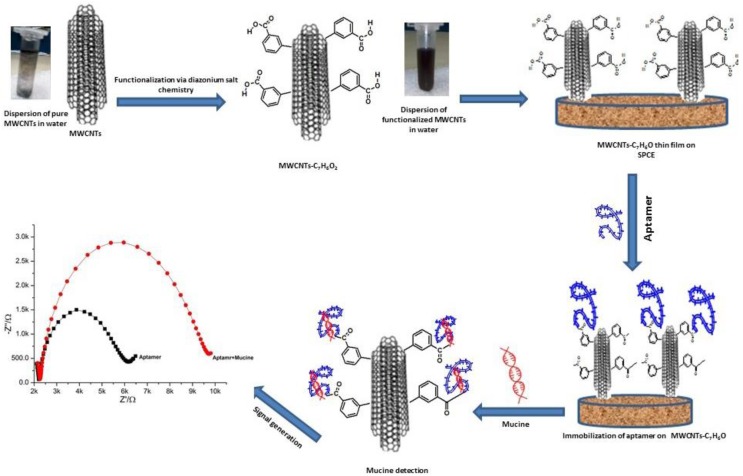
Schematic presentation of thin film assembly of carbon nanotubes on screen printed interface for electrochemical aptasensing applications.

The schematic presentation and the detection principle of the designed aptasensor were provided in Figure 5.

### 3.2. Optimization of the Analytical Parameters for Mucine Detection

Prior to mucine detection, the aptasensor was optimized for experimental conditions such as concentration of aptamer and incubation time of mucine. Concentration of aptamer is an important factor in aptasensor design to achieve a better response against a target analyte, so different concentrations of aptamer were evaluated to find the optimum response against mucine. The electrochemical signal reached a saturation level with an aptamer concentration of 2 µM for detection of mucine (Figure S3a). For best analytical performance of aptasensor, incubation time of analytes is also very important. Electrochemical responses were evaluated for varying incubation times (5, 15, 30, 45 and 60 min) of mucine. Optimal incubation time of mucine was 45 min with mucine–aptamer response reaching equilibrium at 45 min (Figure S3b).

### 3.3. Impedimetric Detection of Mucine

**Figure 6:**
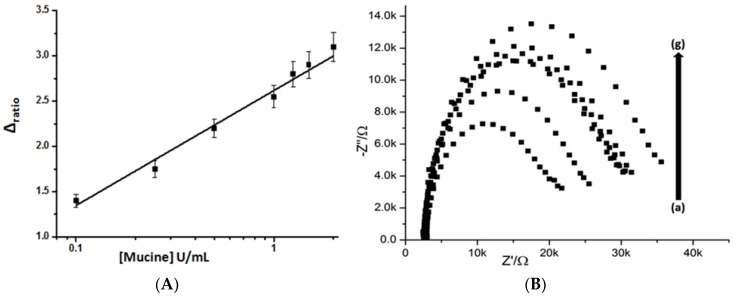
The calibration curve corresponding to the detection of mucine based on changes of electron-transfer resistance (Ret), which presented as ∆ ratio (**A**) Nyquist plots of mucine-aptamer modified SPCEs with different concentrations of mucine U/ml (a) 0.1 (b) 0.25 (c) 0.5 (d) 1 (e) 1.25 (f) 1.5 and (g) 2 (**B**).

After optimization of experimental parameters, an EIS aptasensor was employed for the quantitative analysis of mucine. R_et_ values were calculated and presented as ∆ ratio in the graph. The calibration curve and linear range for mucine detection were presented in Figure 6A. Experimental results show that net R_et_ increased as the mucine concentration increased, originating from the binding of aptamer modified electrode surface with the bulky mucine molecules. The original impedimetric signals are also shown in Figure 6B as Nyquist plots. The specific mucine–aptamer interaction induced an increase of ∆ ratio. A very low limit of detection (0.02 U/mL) with a linear range from 0.1 U/mL to 2 U/mL was achieved without any additional amplification step. Similarly, the observed dissociation constant of the assays was 0.63 µM, which is approximately equal to 0.1 U/mL. An analytical performance comparison of the proposed aptasensor with previously reported electrochemical sensors for detection of MUC1 is provided in Table 1.

### 3.4. Specificity of Mucine Aptasensor

For practical applications, the specificity of aptasensor for target molecules is also highly desired for diagnostic sensors. A sensor will be considered reliable if it does not generate a signal for nonspecific molecules. Therefore, the specificity of the developed aptasensor was evaluated by performing control experiments using nonspecific binding proteins, including BSA, FBS and lysozymes. The MWCNT modified electrodes were incubated for 45 min with these nonspecific proteins. Figure S4 shows the changes of electron-transfer resistance (∆R_et_) of the MWCNT modified SPCE after incubation with BSA, FBS, lysozyme and mucine. From this figure, it can be seen that the ∆R_et_ values for nonspecific proteins are much smaller than those for mucine. These results illustrate that the effect of nonspecific proteins is negligible on mucine detection, and the proposed aptasensor has sufficient specificity to mucine.

### 3.5. Demonstration of the Aptasensor for Clinical Diagnosis

For clinical application of the developed sensor to the diagnosis of mucine, it was essential to investigate its performance for real sample analysis. Herein, performance of the aptasensor was evaluated in human blood serum, one of the most challenging media, which contains complex components. Standard addition methods were used to detect MUC1 spiked in human serum. During the process, three samples were prepared by adding different concentrations of mucine (0.1 U/mL, 0.5 U/mL and 1 U/mL) to 10-fold-diluted healthy human serum obtained from the Shaukat Khanum Memorial Cancer Hospital & Research Centre. The aptasensors were incubated using the same protocol described earlier. Each measurement was performed in triplicate, and then the averaged mucine concentrations were determined from the calibration curve. The data indicates acceptable results of the recoveries and relative standard deviation values for the proposed aptasensor (Table S2). These results clearly suggested that the proposed aptasensor can be used to detect MUC1 in real samples with good reproducibility and sensitivity. The developed aptasensor can be a promising feature for the clinical diagnosis in complex biological samples.

## 4. Conclusions

In summary, we have demonstrated a very simple and inexpensive way to obtain thin films of CNTs onto a screen printed transducer interface. Fabrication of a highly selective monolayer with a high density of CNTs in small features with complex shapes on screen printed transducers provided an ideal platform for on-surface chemistry, and thus has been demonstrated in the construction of an electrochemical aptasensor. This proposed assembly method offers various potential advantages in the construction of biosensors such as no need of any prior chemical modification of carbon electrode surface, use of water as solvent, one step simple fabrication directly from solution using a very small amount of CNTs, and reuse of solution used for thin film assembly. Although the designed surface is demonstrated in the design of the electrochemical aptasensor, it can find widespread application in the fields of sensing and biosensing, and can be very easily extended to the designing of other types of bioreceptor surfaces such as those based on enzymes, antibodies, or cells such as recognition elements.

## Figures and Tables

**Table 1:** Analytical performance of the proposed aptasensor with the previously reported electrochemical sensors for detection of MUC1.

Sr. No	Method Principal	LOD	Linear Range	Ref.
1	“Signal-on” electrochemical aptasensor	0.33 nM	1–20 nM	[24]
2	Immobilization of redox-labeled hairpin DNA aptamers on gold	50 nM	1.50 µM	[54]
3	Aptasensor based on enzyme–gold nanoparticle dual label	2.2 nM	8.8–353.3 nM	[55]
4	Electrochemical immunoassay based on aptamer–protein interaction	0.62 ppb	1–12 ppb	[56]
5	Sandwich format based magnetic beads coupling screen-printed arrays	0.07 nM	0–0.28 nM	[57]
6	Impedimetric aptasensor based on gold nanoparticles	0.1 nM	0.5–10 nM	[58]
7	Insertion approach electrochemical aptasensor based on exonuclease-assisted target recycling	4 pM	10 pM–1 μM	[59]
8	dual signal amplification of poly(o-phenylenediamine) carrier and functionalized carbon nanotube tracing tag	1 pM	1–100 nM	[60]
9	Carbon nanotube thin film assembly on Screen Printed Interface	0.02 U/mL	0.1–2 U/mL	Present work

Mucine (MUC1).

## Supplementary Materials

The following are available online at www.mdpi.com/1424-8220/16/10/1651/s1.
