# Supplementary material for: One Step Assembly of Thin Films of Carbon Nanotubes on Screen Printed Interface for Electrochemical Aptasensing of Breast Cancer Biomarker
Source: Sensors (Basel). 2016 Oct 6;16(10):1651. doi: 10.3390/s16101651 (PMC5087439; doi:10.3390/s16101651)
Supplement: Supplementary file 1 [file sensors-16-01651-s001.pdf]

Supplementary Materials: One Step Assembly of Thin Films of Carbon Nanotubes on Screen Printed Interface for Electrochemical Aptasensing of Breast Cancer Biomarker

Muhammad Azhar Hayat Nawaz [1], Sajid Rauf [1], Gaelle Catanante [2], Mian Hasnain Nawaz [1], Gilvanda Nunes [3], Jean Louis Marty [2,*] and Akhtar Hayat [1,*]

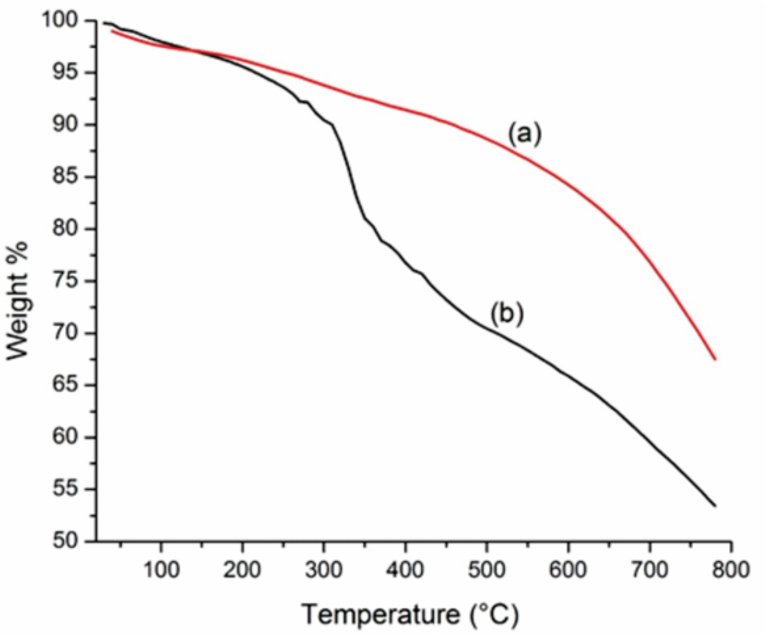

Figure S1. TGA curves of pure CNTs (**a**) and functionalized CNTs (**b**).

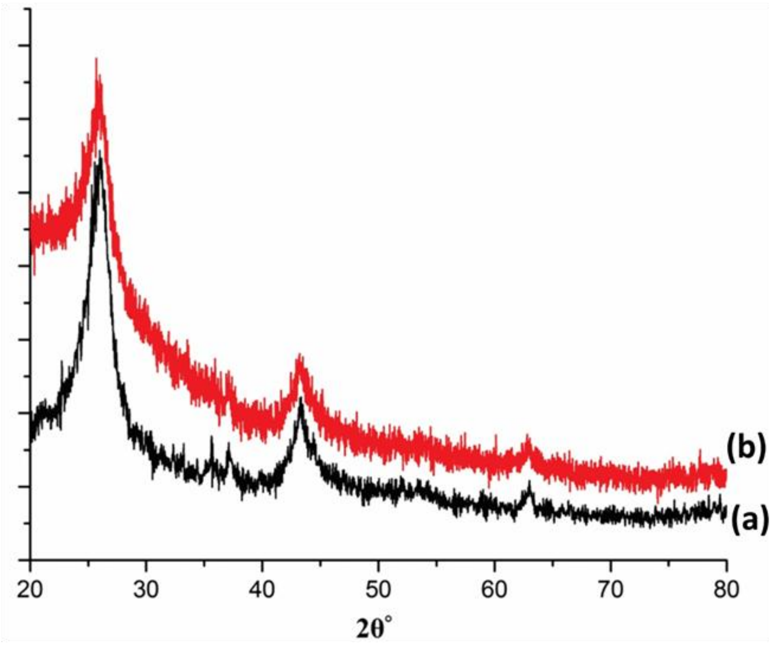

Figure S2. X-ray diffraction (XRD) patterns of pure CNTs (**a**) and functionalized CNTs (**b**).

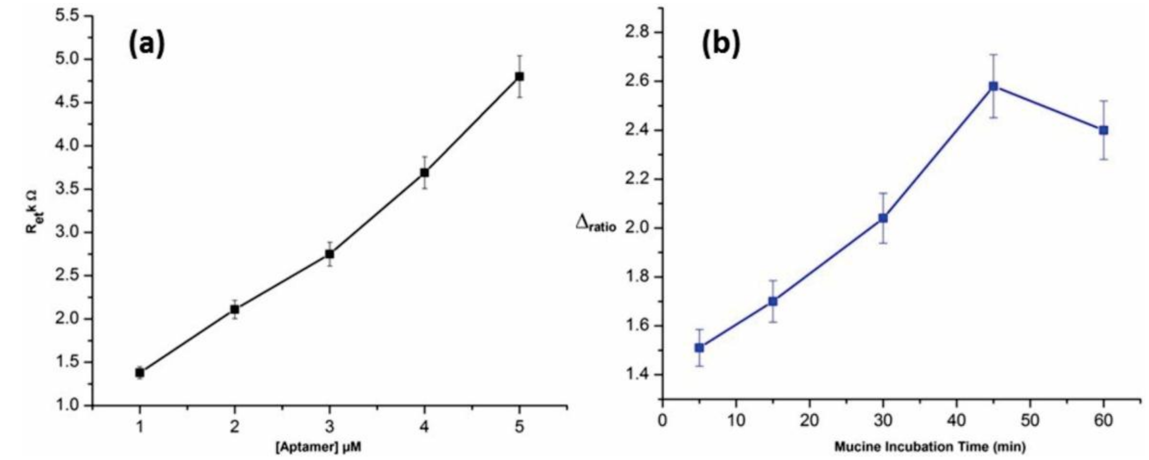

Figure S3. (**a**) Optimization of the concentration of aptamerfor mucine detection (**b**) incubation time period of mucine forthe construction of impedimetricaptasensor.

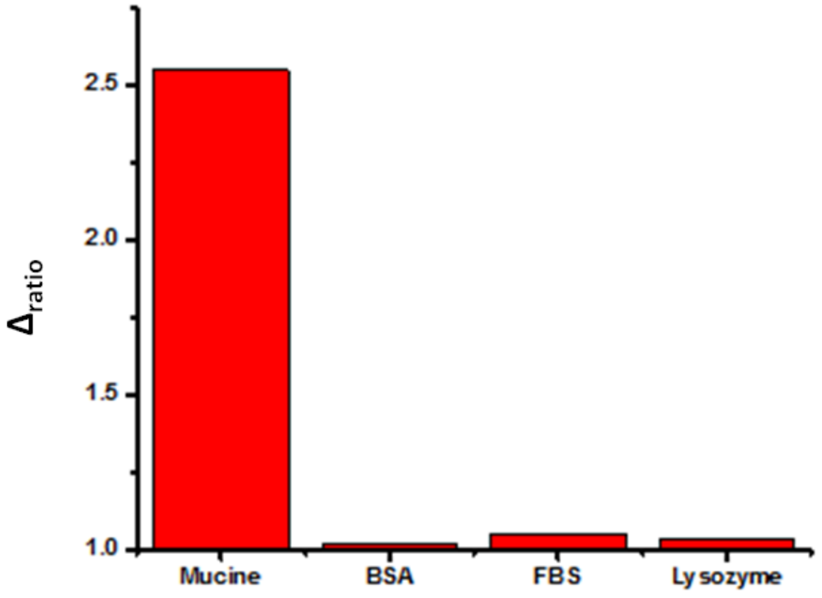

Figure S4. Selectivity analysis of the designed aptasensor under similar experimental conditions.

Table S1. Stimulated values of all elements in the equivalent circuit for the various steps of the aptasensor fabrication and the reaction between aptamer and target analyte.

Electrode	R_s (kΩ·cm^2)	R_{et} (kΩ·cm^2)	Q (µF)/n	10^3 W
SPCE	0.802	7.5	0.930/0.92	1.15
SPCE/MWCNT	0.806	8.3	1.5/0.89	0.163
SPCE/MWCNT/EDC	0.812	1	1.13/0.91	0.089
SPCE/MWCNT/EDC/Apt	0.810	1.5	1.33/0.94	0.156
SPCE/MWCNT/EDC/Apt/Mucine	0.811	3	1.26/0.90	0.143

Table S2. Recovery percentages obtained with designed electrochemical aptasensor.

Mucine Added (U/mL)	Mucie Found (U/mL)	R.S.D %	R.E %	R %
0.1	0.096	3.2	4	96
0.5	0.49	3.8	2	98
1	0.95	4.1	5	95

R.S.D % = relative standard deviation percentage; R.E % = relative error percentage; R % = recovery percentage.
